# Editorial: Psychological Safety in Healthcare Settings

**DOI:** 10.3389/ijph.2024.1608073

**Published:** 2024-12-02

**Authors:** José Mira, Andrea Madarasova Geckova, Bojana Knezevic, Paulo Sousa, Reinhard Strametz

**Affiliations:** ^1^ Alicante-Sant Joan Health Disctrict, Fundación para el Fomento de la Investigación Sanitaria y Biomédica de la Comunidad Valenciana (FISABIO), Alicante, Spain; ^2^ Health Psychology Department, Miguel Hernández University of Elche, Elche, Spain; ^3^ European Researchers’ Network Working on Second Victims (ERNST), COST Action 19113, Brussels, Belgium; ^4^ Department of Health Psychology and Research Methodology, University of Pavol Jozef Šafárik, Košice, Slovakia; ^5^ Institute of Applied Psychology, Comenius University, Bratislava, Slovakia; ^6^ Department for Quality Assurance and Improvement in Healthcare, University Hospital Centre Zagreb, Zagreb, Croatia; ^7^ NOVA National School of Public Health, Public Health Research Centre, Comprehensive Health Research Centre, CHRC, Universidade Nova de Lisboa, Lisbon, Portugal; ^8^ Wiesbaden Business School, RheinMain University of Applied Sciences, Wiesbaden, Germany

**Keywords:** psychological safety, second victim, healthcare workers, quality of care, safety culture

Patient safety is a priority in all healthcare systems. Despite this, up to 24% of hospital admissions and around 7% of primary care patients experience adverse events (AEs) annually, with approximately 50% being preventable [1, 2]. In the EU alone, these preventable AEs result in a loss of 1.5 million disability-adjusted life years (DALYs) and a cost of 19.53–43.65 billion euros in 2024 [3], with a significant impact on the quality of care.

Most of these preventable AEs are due to suboptimal working conditions [4]. Uncertainty, overload, fatigue, and complexity are common limiting factors for quality care, including patient safety. Healthcare workers often face psychological trauma from events such as life-threatening incidents, needle sticks, dramatic deaths, violence, patient deterioration, resuscitations, complaints, suicidal tendencies, and errors causing patient harm. These can alter the practice and morale of healthcare workers, impacting patient outcomes. Therefore, workforce resilience is key to providing optimal care. Otherwise, when overwhelmed and lacking coping resources, they become second victims [5]. They are “any healthcare worker directly or indirectly involved in an unanticipated adverse patient event, unintentional healthcare error, or patient injury, who becomes victimized in the sense that they are also negatively impacted.”

Organizational factors and personality traits influence the second victim experience. Providing safe working conditions is part of the WHO’s objectives for safer care [6]. Professionals must feel supported, trained, equipped, protected, rested, and provided with a suitable work environment, reducing the intensity of this experience as second victims. Addressing this involves healthcare authorities, health professions, scientific societies, academia, patient associations, and civil society and requires a commitment to self-care, prevention programs, and emotional support interventions.

Safety culture, particularly Psychological Safety, is crucial. Introduced by Amy Edmondson [7] in 1999, it describes the ability to speak without fear about performance, including mistakes, to improve care. Without this, patient safety is at risk [8, 9]. However, the blame culture remains prevalent in healthcare [10], impacting how professionals address safety incidents. Fear of blame hinders progress toward a safety culture. Many institutions comply with WHO’s safe practices but fail to engage professionals in patient safety, reacting to dramatic events without preventing potential harm. Proactive risk management fosters a culture of safety. These organizations are on the verge of sharing a culture that generates safety (Figure 1).

**FIGURE 1:**
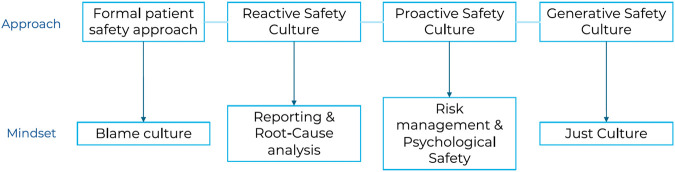
Patient safety path: from formal culture to generative culture of patient safety (Europe, 2024).

Since healthcare workers are not adequately trained to warn colleagues of risky behavior, manage reactions, or support second victims (Kupkovicova et al.; Carrillo et al.) [11], educational reforms are needed to address identified educational gaps in patient safety and to integrate second victim support into the training of medical, nursing, and other healthcare students. Equipping future professionals with skills to recognize and address the second victim phenomenon fosters a supportive work environment and improves patient safety outcomes. Ultimately, these changes can lead to improved quality of care, better patient safety outcomes, and a more resilient healthcare workforce.

To support healthcare professionals and prioritize patient safety and wellbeing, organizations must:

1. Create a fair and accountable environment: Implement policies ensuring transparency and fairness in evaluating performance and handling errors, fostering trust and openness.
2. Balance safety and accountability: Understand root causes of errors and address systemic issues to prevent recurrence, balancing individual accountability with systemic improvements.
3. Commit to continuous improvement and transparency: Regularly evaluate safety protocols, using incident data to drive change, and promote openness to build trust.
4. Learn from incidents: Analyze incidents, identify contributing factors, and develop risk mitigation strategies, empowering staff to participate in safety initiatives.
5. Promote fairness in incident response: Distinguish between honest mistakes, at-risk behavior, and reckless behavior, focusing on system-wide improvements and creating a supportive environment.

By implementing these strategies, healthcare organizations can better support professionals and cultivate a just culture, benefiting patients. Encouraging self-care, resilience, and emotional support, along with fairness and continuous improvement, creates a more effective and compassionate healthcare system.

## References

[1] Bates DW Levine DM Salmasian H Syrowatka A Shahian DM Lipsitz S The Safety of Inpatient Health Care. N Engl J Med (2023) 388(2):142–53. PMID: 36630622. 10.1056/NEJMsa2206117 36630622

[2] Panagioti M Khan K Keers RN Abuzour A Phipps D Kontopantelis E Prevalence, Severity, and Nature of Preventable Patient Harm Across Medical Care Settings: Systematic Review and Meta-Analysis. BMJ (2019) 366:l4185. PMID: 31315828; PMCID: PMC6939648. 10.1136/bmj.l4185 31315828 PMC6939648

[3] Agbabiaka TB Lietz M Mira JJ Warner B. A Literature-Based Economic Evaluation of Healthcare Preventable Adverse Events in Europe. Int J Qual Health Care (2017) 29(1):9–18. PMID: 28003370. 10.1093/intqhc/mzw143 28003370

[4] Hickam DH Severance S Feldstein A Ray L Gorman P Schuldheis S The Effect of Health Care Working Conditions on Patient Safety: Summary. In: AHRQ Evidence Report Summaries, 74. Rockville (MD): Agency for Healthcare Research and Quality (US) (1998-2005) (2003). Available from: https://www.ncbi.nlm.nih.gov/books/NBK11929/ (Accessed July 30, 2024). PMC4781355 12723164

[5] Vanhaecht K Seys D Russotto S Strametz R Mira J Sigurgeirsdóttir S An Evidence and Consensus-Based Definition of Second Victim: A Strategic Topic in Healthcare Quality, Patient Safety, Person-Centeredness and Human Resource Management. Int J Environ Res Public Health (2022) 19(24):16869. 10.3390/ijerph192416869 36554750 PMC9779047

[6] World Health Organization. Global Patient Safety Action Plan 2021-2030: Towards Eliminating Avoidable Harm in Health Care. Geneva (2021).

[7] Edmondson A. Psychological Safety and Learning Behavior in Work Teams. Adm Sci Q (1999) 44(2):350–83. 10.2307/2666999

[8] Pacutova V Geckova AM de Winter AF Reijneveld SA. Opportunities to Strengthen Resilience of Health Care Workers Regarding Patient Safety. BMC Health Serv Res BMC Health Serv Res (2023) 23(1):1127. PMID:37858175. 10.1186/s12913-023-10054-0 37858175 PMC10588085

[9] Dietl JE Derksen C Keller FM Lippke S. Interdisciplinary and Interprofessional Communication Intervention: How Psychological Safety Fosters Communication and Increases Patient Safety. Front Psychol (2023) 14:1164288. PMID: 37397302; PMCID: PMC10310961. 10.3389/fpsyg.2023.1164288 37397302 PMC10310961

[10] van Marum S Verhoeven D de Rooy D. The Barriers and Enhancers to Trust in a Just Culture in Hospital Settings: A Systematic Review. J Patient Saf (2022) 18(7):e1067–e1075. Epub 2022 May 19. PMID: 35588066. 10.1097/PTS.0000000000001012 35588066

[11] Sánchez-García A Saurín-Morán PJ Carrillo I Tella S Põlluste K Srulovici E Patient Safety Topics, Especially the Second Victim Phenomenon, Are Neglected in Undergraduate Medical and Nursing Curricula in Europe: An Online Observational Study. BMC Nurs (2023) 22(1):283. PMID: 37620803; PMCID: PMC10464449. 10.1186/s12912-023-01448-w 37620803 PMC10464449

